# Empathy-Based Training in a Geriatric Workforce Enhancement Program (GWEP) for Dementia Care

**DOI:** 10.1093/geroni/igaf122.3922

**Published:** 2025-12-31

**Authors:** Guillermina Solis, Emre Umucu, Andrew Vernon, Beatrice Lee, Cecilia Fierro, Hyejin Jung

**Affiliations:** The University of Texas at El Paso, El Paso, Texas, United States; The University of Texas at El Paso, El Paso, Texas, United States

## Abstract

In the southwest border region, formal dementia training has traditionally been limited, and educational resources and community support for care have been scarce. As a first-year Geriatric Workforce Enhancement Program (GWEP) recipient of a HRSA grant, the goal is to enhance training and educational resources for the formal and informal healthcare workforce in the region. Programs, such as Dementia Live®, that align with provision of content within age-friendly and dementia friendly care are selected to address the critical gap of knowledge as an entry point into dementia care education for diverse disciplines and caregivers. The initiative implemented Dementia Live® training, which provides an immersive, empathy-based experience to 119 Hispanic/Latino participants, who held various care roles in a variety of health care settings. The effectiveness of the training was evaluated with a pre and post training survey and statistical comparison with paired t-test was conducted to examine changes in dementia knowledge. The results showed a statistically significant increase in knowledge scores from pre-training (M = 3.29) to post-training (M = 3.81), t(118)=5.92, p<.001. Additionally, a moderate effect size (d = 0.54) was observed indicating a meaningful practical impact. This study supports that an empathy-focused educational approach is highly effective in boosting dementia knowledge among multidisciplinary care teams. By reaching a broad, underserved population, this project demonstrates a replicable model for GWEP initiatives to build geriatric care readiness and establish essential educational and community support infrastructure in areas lacking resources. A key strength of this initiative was its ability to engage a diverse workforce.

